# Animal models of developmental dyslexia

**DOI:** 10.3389/fnins.2022.981801

**Published:** 2022-11-14

**Authors:** Albert M. Galaburda

**Affiliations:** Beth Israel Deaconess Medical Center, Harvard Medical School, Boston, MA, United States

**Keywords:** animal models, developmental dyslexia, brain development, endophenotypes, brain asymmetry, ciliopathies, brain anomalies

## Abstract

As some critics have stated, the term “developmental dyslexia” refers to a strictly human disorder, relating to a strictly human capacity – reading – so it cannot be modeled in experimental animals, much less so in lowly rodents. However, two endophenotypes associated with developmental dyslexia are eminently suitable for animal modeling: Cerebral Lateralization, as illustrated by the association between dyslexia and non-righthandedness, and Cerebrocortical Dysfunction, as illustrated by the described abnormal structural anatomy and/or physiology and functional imaging of the dyslexic cerebral cortex. This paper will provide a brief review of these two endophenotypes in human beings with developmental dyslexia and will describe the animal work done in my laboratory and that of others to try to shed light on the etiology of and neural mechanisms underlying developmental dyslexia. Some thought will also be given to future directions of the research.

## Introduction

There are many doubts associated with the concept of animal models of human biology and disease, ranging from questioning the degree of molecular, cellular, and higher order homology, to the generalizability and translational potential of animal experimentation to human disease, to ethical considerations regarding animal experimentation, each worthy of serious discussion. These caveats clearly apply to animal models of reading disorder, but I hope to show in this partial review that research findings from animal models of reading disorders still have the potential to shed light on causality, mechanisms, early diagnosis and prevention, and on the design of successful therapies.

In the range of biological levels of representation, from genes and molecules, cell biology and circuits, networks, whole brains and organisms, to cognitive and social systems, non-human and human animals differ least at the first level – genes and molecules – and most at the last – cognitive and social systems. This is illustrated by the example that one can use the same bricks to build schools, supermarkets and post offices, each with very different form and use. One would then be permitted to conclude that animal models are *a priori* most reasonable to pursue for understanding the shared smallest biological units—molecules and cells. Of course, such a conclusion would discourage animal models for understanding reading disabilities, because, although reading acquisition certainly depends on the molecular and cellular integrity of the brain, it cannot happen without the appropriateness and health of higher level structures, such as whole brains and organisms, and social constructs. We understand that social constructs are important because, even as the human brain learns many skills spontaneously, or by imitation, in most cases reading has to be taught, which implies the presence of social structures, including family, teachers and schools, none of which can be modeled in animals. All of this would lead to the conclusion that merely understanding the state of molecules, cells, and circuits is not enough for understanding reading or reading disorders; it is also likely that this situation will not change in the near future, if ever. So, on first inspection, there exist grave restrictions on the utility of animal models for reading and dyslexia. One way out of this conundrum is to focus on preadapted structures and behaviors that are indeed present in animals and humans, which comprise necessary, even if not sufficient, building blocks for the cognitive functions seen only in humans. When these preadapted structures are considered in the genetic context, they are called “endophenotypes” (Gottesman and Gould, 2003).^1^

Endophenotypes studied in animal models have requirements. They must be proven to be reasonable facsimiles of the situation in the human (for a review, see Shanks et al., 2009). This means that at some point it must be shown that the results obtained in the animal are comparable to those that would be obtained in the human were the experiments be able to be performed in the human, and that predictions that come true in the animal model will, within reason, come true for the human. For some questions, this may be a particular challenge in mouse studies, given their phylogenetic distance from the human. For instance, identical or near identical genomic homologies in the mouse and human need not predict for equivalent phenotypes or disease states, as modulation or compensation from other genes or environments in the animal may not be available to the human, or *vice versa*. Thus, it is not uncommon to see that a drug that works in the mouse model fails to work in a human clinical trial (Perrin, 2014). Development and aging have such different time courses in rodents and humans that absence of pathology in the mouse is no guarantee that pathology will not eventually emerge in the human. Also, it may not be possible to mimic all aspects of a disease state in small animals with strikingly different developmental histories, for instance being raised in a mouse cage without social contact, where early experiences can modify the expression of the endophenotype in question (Denenberg, 1981). Here is where the appropriate selection of endophenotypes helps. In that case, a well-chosen endophenotype can shed light on the pathophysiology of the human disorder and can provide ideas for non-invasive testing in the human. In the case of developmental disorders, such as dyslexia, the issue of non-invasive testing becomes particularly important, since often one is dealing with children.

## Endophenotypes to model

What makes most sense to model are endophenotypes that *a priori* are more likely to be equivalent in animals and humans. These might include molecular pathways and cellular functions associated with shared dyslexia risk genes, genes that have homologies in both species. Here, even at this low level of representation, care must be taken not to freely generalize from one species to another, since effects of gene manipulation may vary across species and according to the methods used to manipulate gene expression. Based on the known neuroanatomical abnormalities and cognitive deficits in individuals with dyslexia, some preadapted sensory and perceptual behaviors involving the visual and auditory systems, or attention and memory, or laterality, for instance, could comprise suitable behavioral endophenotypes. In our laboratories we chose endophenotypes guided by the original findings in dyslexic autopsy brains—neuronal migration anomalies and anomalous brain asymmetries (Galaburda and Kemper, 1979; Galaburda et al., 1985), which generated additional behavioral research in the animals (see, c.f., Fitch et al., 1994, 1997; Clark et al., 2000a,b).

The first descriptions of structural changes in the brain of dyslexic individuals were described in the 1970s and 1980s on a few dyslexic individuals who had died of unrelated causes (Galaburda and Kemper, 1979; Galaburda et al., 1985, 1994; Humphreys et al., 1990). They ranged in age from the 30s through the 80s, none of them children, and they comprised both men and women. The extent to which the diagnosis was confirmed in life varied, being less secure in the aged individuals. Two types of findings stood out: (1) Subtle disturbances in cortical development, called layer 1 heterotopias, subpial heterotopias, or, simply, ectopias; and (2) abnormal asymmetry of the planum temporale, a region on the upper surface of the temporal lobe associated with language function. In women, the lesions were somewhat later in development and consisted of small, myelinate intracortical scars in the same distribution as the males (Humphreys et al., 1990). In addition, the standard pattern of a left larger planum temporale, seen in two thirds of control human brains (Geschwind and Levitsky, 1968), was not found in any of 7 the dyslexic brains. Attempts were made to confirm the anatomical findings in additional *post-mortem* brains, but this proved to be impossible. Funding to harvest brains in a condition that begins in childhood and normally does not lead to death was simply not forthcoming, and the autopsy project had to be abandoned. Furthermore, the microscopic developmental cortical anomalies, measuring only a few hundred micra in diameter, were not amenable to *in vivo* imaging because the imaging equipment lacked the spatial and contrast resolution needed, although larger structural gray matter heterotopias that are causally related to the smaller anomalies seen in the autopsy brains, have been imaged (Chang et al., 2005). In fact, the difficulty in demonstrating microscopic developmental cortical anomalies in living research participants served as an important stimulus for developing animal models. Another, and perhaps more important reason, was that animal models could be manipulated to test hypotheses about fundamental causes in ways not practically or ethically possible in human studies.

There is a literature about non-right handedness (Abbondanza et al., 2022) and right hemisphere activation (Pugh et al., 2000) for language tasks in dyslexic populations that indicate an aberration in cerebral dominance. Also, a few studies have been published on the issue of structural planum temporale asymmetry as seen in *in vivo* imaging studies (for a review, see Shapleske et al., 1999). In the autopsy studies published by Galaburda and colleagues, the planum temporale was uniformly symmetric, while in the classical study by Geschwind and Levitsky (1968), only 24% of the sample of 100 normal adult human brains showed symmetrical plana. *In vivo* imaging has produced differing results on this topic, which mainly results from slight differences in defining the borders of the planum temporale. One study, that of Altarelli et al. (2014), which defined the planum identically to Geschwind and Levitsky, albeit from MR image reconstructions, rather than from photographs of the upper surface of the temporal lobe in autopsy specimens, found that the asymmetry pattern in the planum of dyslexic brains differed from controls, but only in dyslexic boys, who showed a greater proportion of rightward asymmetrical cases^2^. Also, Heschl’s gyrus, which is sometimes duplicated on the right side in control brains, is significantly more often duplicated in dyslexic boys. The difference between the Geschwind and Levitsky and the Altarelli et al. (2014) findings is not understood, although both findings support an anomaly in the manifestation of asymmetry of a language area in the dyslexic brain, at least in boys and men. And, at least in boys, there is a deviation in the distribution of planum asymmetry, which, together with reports of an abnormal distribution of hand dominance in the dyslexic population (Abbondanza et al., 2022), make modeling asymmetry and laterality in animals potentially productive.

Non-human animals display individual paw or claw preference and a directional preference for body rotation and circling behavior. For instance, caged rats will preferentially hug the right or the left wall when exploring the cage (Denenberg, 1981; Glick and Ross, 1981), but show less of a population bias than humans (Glick and Ross, 1981). On the other hand, humans display a strong tendency to right handedness at the population level. This tendency to right-handedness is matched to a large extent by left-hemisphere dominance for speech and language (Knecht et al., 2000). The mechanisms of handedness and language lateralization are not known, but cilia may play a role. Cilia are short, microscopic, hairlike cellular structures that are responsible for the left-right body patterning that results in a left sided-heart and a right sided liver, for instance (Dasgupta and Amack, 2016), but do not easily explain brain laterality (Trulioff et al., 2017). Cilia can be rendered dysfunctional by suppressing the expression of some of the dyslexia-associated genes, and, as they are conserved between humans and animals, they can comprise a useful endophenotype to model in the study of dyslexia.

In addition to asymmetries and developmental cortical malformations, there exist behavioral characteristics displayed by individuals with dyslexia that could also be amenable to animal modeling. Thus, even though the reading disability *per se* cannot be modeled, for obvious reasons, there are some sensory-perceptual traits underlying reading that can be. So, for instance, dyslexic persons have been shown to exhibit phonological deficits as a result, at least in part, of abnormal sound processing at levels lower than the cognitive and cortex (Hornickel and Kraus, 2013; Neef et al., 2017). The idea is that if sounds are not processed properly, which includes the processing of speech relevant sounds, then abnormal phonological representations arise, which represent a barrier to learning to read easily. Phonology is a complex term that includes both speech sound representations (phonetics) and phonological grammar (i.e., rules for combining a limited number of speech sounds to produce unlimited words and meanings). It appears that at least the phonetics part of phonology lends itself to investigation in animals; for instance, it was shown more than fifty years ago that chinchillas can be taught to make speech sound distinctions (Kuhl and Miller, 1971). Furthermore, the phonological grammar appears to be spared in dyslexia (Berent et al., 2012, 2013).

## Animal models of dyslexia

### Neonatal freezing injury model

Animal studies in this field have exploited the freezing injury rat model, the short hairpin RNA interference (shRNAi) rat model, and the gene deletion (knockout) mouse model. A non-exhaustive review of these studies is presented below for the purpose of illustrating the kinds of discoveries that can be made from using such models.

The neonatal freezing injury model consisted in placing lesions in the developing cortex during the neonatal period, before neuronal migration to the cortex ends. Initially, we used rats prepared by the method established by Dvorak, Feit and Juránková (Dvorak et al., 1978). First, on day one or two after birth, when neuronal migration to the cerebral cortex is still proceeding but reaching the end as the upper layer neurons finish their migration, we apply a freezing probe to the skull of the newborn. Depending on the duration of the probe application, a molecular layer ectopia, a 4-layer microgyrus, or frank porencephaly is produced (Humphreys et al., 1991; Suzuki and Choi, 1991; Rosen et al., 1992, 2000). The coexistence of these very different-looking abnormalities has been recognized in abnormal human brain development for many years (Friede, 1989), so these malformations are considered causally linked. Although molecular layer ectopias were the main finding in the autopsied dyslexic brains, there were also a couple of instances of microgyria, but no instances of porencephaly; porencephaly in the perisylvian language cortex is a more severe lesion and would more likely present with speech and/or language delay and epilepsy as part of the perisylvian syndrome (Kuzniecky et al., 1993). It is not farfetched, then, to hypothesize that microgyria and molecular layer ectopias, by virtue of being a milder pathology, would be associated with more subtle cognitive deficits, *e.g.*, dyslexia, and techniques are available to mimic these pathologies in the developing rat.

Rats with freezing lesions^3^ start as normal animals. Starting with a normal animal, anything done to it in the laboratory represents the initial event, thus the cause of what happens subsequently. It is important to stress that the brain reacts to the initial event by a process known as plasticity.^4^ However, the reaction to the initial event need not make things better, and anatomical, physiological, and behavioral abnormalities documented later may be the result of this plasticity plus the initial event, rather than the initial event alone, thus making the plasticity potentially a maladaptive rather than an ameliorative phenomenon. This negative effect may be particularly true for very early lesions, in violation of the so-called Kennard Principle (Schneider, 1979; Johnston, 2004; Elliott, 2020). In the case of the rat with the freezing lesion, the injury triggers plasticity in connections and in the cell composition of connectionally related areas (Rosen et al., 1998; Li et al., 2021). In rats with shRNAi and in the knock-out mouse, epigenetic changes are triggered in other genes that are part of the injured gene’s network (Che et al., 2014, 2016). The rat freezing injury model has the additional advantage that it has available to it a larger repertoire of behaviors that can be tested in the laboratory, as compared to a substantially reduced repertoire in the mouse. Working with the rat, whether in a freezing injury model or using shRNAi gene knockdown, affects development during late neuronal migration to the neocortex, whereas the gene deletion in the mouse knockout is earlier and precedes neuronal migration. Thus, in addition to the species-specific differences, there is a developmental timing difference that needs to be taken into consideration when interpreting differences in outcomes. That said, even though the freezing injury rat model illustrates the enormous plasticity of the developing brain, there has never been any evidence that dyslexia in humans arises from an episode of intrauterine brain injury, whether traumatic, vascular, infectious, metabolic or other. Instead, there has been growing evidence that variants of certain genes that are expressed in the brain during development contribute significant risk for dyslexia. Therefore, when genetic epidemiological studies began to identify these risk genes, our laboratories retooled to study them in gene-based animal models.

### Genetics, dyslexia, and animal research

In the past 20 years, several dyslexia risk genes have been discovered around the world. The first of these genes was *DYX1C1*, followed closely by *DCDC2, KIAA0319, ROBO1, EKN1* (Paracchini et al., 2007).^5^ These are called risk, or susceptibility, genes, because they do not predict for a particular phenotype or disorder to arise, but rather for the risk that it will arise. Their discovery involves large scale population studies and statistical associations between the presence of a genetic marker on a chromosome and the presence of a phenotype or disorder. After identifying the marker additional work needs be done to identify the gene and the mutation or variant, and still more work to understand its functions. In many, if not the majority of situations, the variant associated with the condition does not involve the protein coding part of the gene, the exon, but rather a regulatory segment, such as a transcription factor that modulates timing and degree of expression of the exon, the so-called epigenetic activity. In many cases, initially it is difficult to see how a particular gene variant or mutation, and its downstream effects, lead to the phenotype of interest, but this discovery is made easier (but not easy!) if the gene in question is expressed in the organ of interest and during the time the science suggests the phenotype originates. In the case of dyslexia, based on what we know about the brain, the gene would at least have to be expressed in the brain during the time of neuronal migration to the cortex, but not necessarily in the developing cortex, since the cortical changes could be secondary to an initial event at other sites. However, it would be surprising, if not embarrassing, to discover that a statistically identified risk gene for dyslexia is only expressed in the liver during senescence!

Genes for dyslexia have effects on human brain development, but it remains a challenge knowing how these effects lead to reading disabilities. The functions of these genes are mainly known from work on cell preparations, rodents, fish, flies and worms, which adds a layer or more of separation from the problem at hand in the human. Furthermore, in general the dyslexia risk genes are broadly expressed in the animal brain and human brain in neurons, so a deficit in a narrow set of cognitive domains, say hearing, vision, language and reading, does not easily follow from such a broad neural distribution, which instead may predict for general intellectual disability, motor and sensory deficits, and/or epilepsy. At the writing of this paper, this conundrum remains an important challenge to the science, but it can be argued that continued work on animal models is likely eventually to provide at least some of the answers (also see below in the discussion section).

### DYX1C1

*DYX1C1* was the first reported dyslexia susceptibility gene (Taipale et al., 2003). *Currently* termed DNAAF4 (dynein axonemal assembly factor 4^6^), this gene encodes a tetratricopeptide repeat (TPR) domain-containing protein. TPR is a broadly occurring structural motif that helps with protein-protein interactions and the assembly of multiprotein structures and has been linked to several disorders, including primary ciliary dyskinesia (Loges and Omran, 2018), whereby cilia are involved in neuronal migration, particularly interneuron migration, although their role in excitatory neuron migration cannot yet be excluded (Guemez-Gamboa et al., 2014). A chromosomal translocation involving *DYX1C1* confers a susceptibility to developmental dyslexia. Multiple, focal neuronal migration abnormalities primarily in the left perisylvian (language) cortex comprised the most prominent finding in several brains of dyslexic individuals studied at autopsy (Galaburda and Kemper, 1979; Galaburda et al., 1985). So, it was particularly reassuring to discover that suppression of *Dyx1c1* protein translation in the rat by short-hairpin RNA interference (shRNAi) in the late fetal period caused neuronal migration anomalies of cortical projection neurons arising in the ventricular zone (Rosen et al., 2007). Clumps of neurons remained in the ventricular zone, while others over-migrated beyond the layers that would normally accommodate them (Currier et al., 2011). Abnormalities were not restricted to the cerebral cortex. In fact, RNAi transfected rats displayed changes in the medial geniculate nucleus (MGN), with a significant shift to smaller MGN neurons (Szalkowski et al., 2013); autopsied dyslexic brains had shown the same findings in the MGN (Galaburda et al., 1994).

The demonstrated role of *Dyx1c1* varies according to experimental condition. Thus, even though shRNAi interference in rats in late gestation causes cerebro-cortical neuronal migration abnormalities, deletion of exons 2-4 of *Dyx1c1* in mice (*Dyx1c1v* knockout mice), which also renders the gene non-functional, albeit earlier in development, soon after fertilization of the ovum, does not (Chandrasekar et al., 2013; Tarkar et al., 2013)*;* also, see below); instead, *Dyx1c1v* knockouts display a phenotype that is reminiscent of human primary ciliary dyskinesia, a disorder characterized by chronic airway disease, laterality defects (*situs inversus*), and male infertility (Lee and Gleeson, 2011; Chandrasekar et al., 2013; Tarkar et al., 2013; Loges and Omran, 2018; Anvarian et al., 2019; Hasenpusch-Theil and Theil, 2021). These knockout mice die soon after birth with hydrocephalus and display situs inversus. Hydrocephalus is an accumulation of cerebrospinal fluid with resultant enlargement of the ventricular system, which implicates dysfunction of the ependymal cell cilia, which are thought to help mobilize the cerebrospinal fluid for resorption (Kumar et al., 2021). In the zebrafish, cilia are present in the Kupffer vesicle (Chandrasekar et al., 2013), which is involved in left-right brain development. However, although cilia are also present in the central nervous system of mammals beyond the empendymal cells, there is no proven relationship between primary cilia dyskinesia and disturbances of cerebral laterality in humans, although in the case of situs inversus without cilia dysfunction, left-handedness has been reported to be increased (Postema et al., 2020). Nevertheless, cilia dysfunction cannot at present clearly explain variations in cerebral asymmetry and increased non-righthandedness among dyslexic individuals. On the other hand, cilia have been implicated in neuronal migration to the cerebral cortex, particularly interneurons migrating tangentially from the ventral germinal zones. Less is known about the radial migration of pyramidal neurons from the ventricular zones, and a portion of patients with the Meckel Gruber Syndrome and Joubert Syndrome, both involving cilia biology, develop heterotopias and other neuronal migration abnormalities (for an excellent review of the role of cilia in neuronal migration, please see Hasenpusch-Theil and Theil, 2021).

One could hypothesize, that cilia dysfunction in dyslexics carrying the *DYX1C1* variant contribute to the neuronal migration defect but also impedes a directional gradient of patterning molecules, which would, in turn, lead to aberrant cerebral asymmetry at the molecular, cellular and perhaps also circuit levels, not yet amenable to demonstration by current *in vivo* tools for human research, let alone in clinical work. In fact, understanding normal and aberrant cerebral asymmetry remains a challenge. Our older studies in rats with experimental cortical microgyria, a type of neuronal migration anomaly described in dyslexia, demonstrated changes in both intra- and interhemispheric connectivity (Rosen et al., 2000), with a theoretical capability of altering patterns of intra and interhemispheric communication, and, thus, lateralization of function. A comparable effect on callosal connections altering lateralization has been suggested for loss of ROBO2 function, another gene implicated in reading disorders in rare families (Hannula-Jouppi et al., 2005). In individuals with dyslexia carrying any of several dyslexia risk genes, the volume of cortical white matter seems to be a predictor of reading comprehension (Darki et al., 2012; Eicher and Gruen, 2013) and alterations in asymmetry of brain activation (Pinel et al., 2012) are seen with the same dyslexia-related polymorphisms. We posit that reorientation of cortical white matter connections in a (seemingly futile) attempt to compensate for the presence of abnormal developmental targets (the malformations), leads to the changes in white matter volumes seen in the imaging studies and in alterations in cerebral lateralization and brain activation during language tasks (but see the glutamatergic hypothesis, below).

### DCDC2

A member of the doublecortin superfamily of genes (Reiner et al., 2006), some of which have been linked to abnormal neuronal migration, epilepsy, blindness, and general intellectual disability, *DCDC2* has also been linked to dyslexia (Meng et al., 2005 and others; but see Scerri et al., 2017). This gene serves as a protein-interaction platform (Reiner et al., 2006), where the doublecortin domain binds tubulin and enhances microtubule polymerization. Microtubules are filamentous intracellular structures that are responsible for various kinds of cell movements, including intracellular transport, axon extension and neuronal migration; microtubules are also implicated in the assembly and signaling of primary cilia. Additional functions of *DCDC2* include dendrite morphogenesis, neuronal action potentials, Wnt signaling, sound perception, and excitatory (glutamatergic) synaptic transmission (Massinen et al., 2011; Che et al., 2016^7^).

Elevated glutamate levels were previously found in attention deficit/hyperactivity disorder (Carrey et al., 2007) and autism (Brown et al., 2013) and have more recently been associated with individual differences in reading ability in young readers (Pugh et al., 2014). Our collaborators showed that *Dcdc2* deletion in mice was accompanied by increased excitability and decreased temporal precision in action potential firing in the cortex (Che et al., 2014, 2016). Furthermore, the decreased action potential temporal precision could be fully restored in mutants by treatment with either the NMDA receptor antagonist (2R)-amino-5-phosphonovaleric acid or the NMDAR 2B subunit–specific antagonist Ro 25-6981 (Che et al., 2014). Precise timing of neuronal firing is likely to be essential for representing speech sounds, some of which require a temporal resolution of only a few milliseconds. A deficit in precise firing could explain a tendency for phonological deficits, on the one hand, and, on the other, absence of other perceptual and cognitive deficits that do not depend on precise, rapid neuronal firing. In this way, a ubiquitous neuronal dysfunction could affect one or a few cognitive/perceptual functions, while leaving others intact. This is a testable hypothesis that can help answer the question of why a dysfunction that can affect most neurons can present with a focal behavioral disorder.

As noted previously, under some experimental conditions, neuronal migration anomalies occur when the function of dyslexia risk gene homologs is suppressed *in utero*. An interesting observation was made when rats were transfected with Dcdc2 shRNA, which silences the gene for a few days. Both undermigration and overmigration of cortical neurons were seen, but, whereas over-migration of transfected neurons occurred with transfection late in the intrauterine period, overmigration did not occur with earlier transfection (Adlerr et al., 2013). This difference suggested that compensation could occur in this endophenotype if the gene silencing was early, but not late. This is à propos of reports, and our own results, this time in *Dcdc2* knockout mice, that failed to show migration anomalies. In the knockout, the gene silencing starts much earlier, and the experiments are carried out in mice instead of rats, where species differences may also play a role. It has also been suggested that *Dcdc2* has a role in neuronal migration only when doublecortin is inhibited, whereby deletion of *Dcdc2* increased the severity of the deficits of neuronal migration caused by RNA interference of doublecortin (Wang et al., 2011).

Human carriers of the rs793842 polymorphism of *DCDC2* show a negative correlation between white matter volume and reading comprehension, as well as thickening of the cortex over the left angular and supramarginal gyri (Darki et al., 2014), areas that participate in language and reading. However, excessive glutamatergic activity or hyperexcitability (see above) would be expected to cause increased excitotoxic apoptosis of neurons and oligodendrocytes leading to cortical atrophy; therefore, the cortical thickening remains unexplained, particularly in the parietal lobes, which are particularly vulnerable to excitotoxicity. Thus, Alzheimer’s disease, a condition associated with excitotoxicity and cell death, shows early atrophic changes in the parietal lobes (Jacobs et al., 2012). The white matter reduction is more easily explained by the special vulnerability of oligodendrocytes to glutamatergic excitotoxicity (see Matute et al., 2007). Increased cortical thickness need not imply better function in a phrenological sense. A thicker cortex can be seen in developmental malformations, such as polymicrogyria (also referred to as “micropolygyria”), in part due to centripetal collapse of 4-layer microgyric cortex and blurring of the cortical-subcortical border and/or decreased developmental neuronal and dendritic pruning. Furthermore, one dyslexia-associated gene variant of *Robo1* causes increased interneuron migration to the cortex (Andrews et al., 2006), which could be another source for the thickening seen, leading to increased intracortical circuits but no increase in longer cortico-cortical pathways. Interneurons’ main neurotransmitter is gamma aminobutyric acid (GABA), which is initially excitatory and trophic and switches to inhibitory later in development, the date determined by the degree of GABA activity and blockade (Ganguly et al., 2001). Significant functional changes in the cortex would then be expected by a process that increases migration of GABAergic interneurons to the cortex. Support for the hypothesis of a thicker albeit dysfunctional cortex comes from MRI activation studies showing that posterior left temporoparietal reading related areas (Meda et al., 2008) activate less strongly during reading tasks (Cope et al., 2012; Eicher and Gruen, 2013; D’Mello and Gabrieli, 2018; Richlan, 2020).

### KIAA0319

KIAA0319 is a transmembrane protein coded by *KIAA0319*, on chromosome site 6p22.2, with relevant expression in the central nervous system, pituitary, and peripheral nervous system (Franquinho et al., 2017^8^). The gene has been extensively studied in human populations *vis à vis* language, reading and cerebral lateralization [see review by Eberli et al. (2021)]. The gene was linked to dyslexia, and its expression was shown to be reduced in individuals carrying a risk haplotype that included *KIAA0319* (Cope et al., 2005; Paracchini et al., 2006). Expression of the other two genes in the haplotype, the TTRAP gene and portions of THEM2, was not reduced, thus pointing the finger to *KIAA0319* (Paracchini et al., 2006). In rat studies, it has been shown that the protein is involved in neuronal migration during cerebro-cortical development *in utero* (Peschansky et al., 2010; Adlerr et al., 2013; Platt et al., 2013; but see Guidi et al., 2017 in mice). *KIAA0319* may function in a cell autonomous and a non-cell autonomous manner and plays a role in appropriate adhesion between migrating neurons and radial glial fibers during neuronal migration (see text footnote 3). It may also regulate growth and differentiation of dendrites. Thus, negative regulation of axon extension and dendrite development has been demonstrated, as well as effect on auditory responses.^9^

Our group used *in utero* electroporation (Peschansky et al., 2010; Platt et al., 2013) to transfect cells in E15/16 rat neocortical ventricular zone with either shRNA vectors targeting Kiaa0319, with a KIAA0319 expression construct, with a Kiaa0319 shRNA along with KIAA0319 expression construct (“rescue condition”), or with a scrambled version of Kiaa0319 shRNA. Knockdown, but not overexpression, of Kiaa0319 resulted in periventricular heterotopias that contained large numbers of both transfected and non-transfected neurons, the latter considered a non-cell autonomous effect on neuronal migration. Of the Kiaa0319 shRNA– transfected neurons that migrated into the cortical plate, most migrated to their appropriate laminae. In contrast, neurons transfected with the KIAA0319 expression vector attained laminar positions subjacent to their expected positions, indicating that both under- and over-expression of the gene affected neuronal migration. Furthermore, neurons transfected with Kiaa0319 shRNA exhibited apical, but not basal, dendrite hypertrophy. The rescue conditions were successful in inhibiting the migrational and dendritic effects of under- and over-expression, which is a method for excluding off-target effects of the transfection. Off-target effects occur when a short vector contains a sequence that is found not only in the target gene, but also in another unknown gene or genes. In that case there is the danger of interpreting the phenotype as resulting from an effect on the target gene, when in fact it results from effects on some unknown gene sharing the same short sequence. Restitution of the known protein by overexpression would work only on the target gene and is a necessary step for excluding off-target effects. On the other hand, comparable effects were not noted in the mouse undergoing gene deletion (mouse knockouts), which led to controversy (Franquinho et al., 2017; Guidi et al., 2017, Guidi et al., 2018; Martinez-Garay et al., 2017): Does *KIAA0319* have anything to do with neuronal migration? For this writer, finding neuronal migration anomalies is more telling than not finding them, when the research has controlled for off-target effects and other artifacts, unless it can be shown that the process for looking for neuronal migration anomalies itself causes them to appear; this has not been shown to be the case in the rats undergoing shRNAi. On the other hand, one can come up with reasons why anomalies may not arise, especially when the counterexample involves an altogether different species and methodology (see above). Here is a situation where animal studies can shed both light and confusion on the real question, which is whether a genetic variant is responsible for a specific endophenotype in humans. Of additional interest is the fact that suppression of gene expression in the *Dcdc2* knockout mouse still produces abnormal cortical physiology, which illustrates the possibility that the neuronal migration anomaly may be only a marker for a more important underlying cortical dysfunction that can exist even in the absence of the marker.

A recent *in vivo* and *post-mortem* study in chimpanzees established a relationship between KIAA0319 variants and gray matter volume in the posterior superior temporal gyrus, as well as neuropil asymmetries in the same region under microscopic examination (Hopkins et al., 2021), suggesting an evolutionary influence by KIAA019 on auditory processing preceding the evolution of language in the primate line. In the absence of linguistic capacities in non-human primates, this effect of KIAA019 supports the notion that dyslexia-related genes are not directed at reading or language *in utero*, but rather to preadapted acoustic endophenotypes that in humans comprise some of the building blocks of language and reading acquisition and efficiency.

In a continuing attempt to make the rodent model as naturalistic as possible, we and others focused on a gene deletion models, known as knock-outs, in the mouse. First, unlike knock-down of gene expression in the rat by shRNAi, deletion of dyslexia risk homologs in the mouse do not result in neuronal migration abnormalities. Instead, deletion of exons 2-4 of *Dyx1c1* in the mouse, which eliminates protein translation, was associated with abnormalities in cilia structure, growth, and function (Chandrasekar et al., 2013; Tarkar et al., 2013). Abnormalities in cilia structure and function were also seen in association with *Dcdc2* dysregulation (Massinen et al., 2011), and a missense mutation in *DCDC2* is known to cause deafness in humans, likely associated with cochlear cilia abnormalities (Grati et al., 2015). Primary ciliopathies are also associated with hearing loss, underscoring the importance of cilia for auditory function. Sonic hedgehog signaling dysregulation causes hearing loss in ciliopathy mouse models (Moon et al., 2020), and Dcdc2 interacts with sonic hedgehog signaling (Massinen et al., 2011). *Kiaa0319* modifications altered axonal growth (Franquinho et al., 2017), and gene overexpression in cortex delayed radial migration, but did not change the pattern of cortical lamination. Similarly, a cell knockout model showed that cilia exhibited increased length and changes in cell migration (Diaz et al., 2022). Finally, *Kiaa0319* knockout animals showed subtle alterations in anxiety-related behavior and in sensorimotor gating (Martinez-Garay et al., 2017).

### Other genes

Other genes have been linked to dyslexia. For instance, ROBO1 affects auditory and visual motion processing that predict for reading achievement (Mascheretti et al., 2020) and vocal learning in animals (Wang et al., 2015); the gene has been associated with increased interneuron migration into the cerebral cortex, as well as altered inter and intrahemispheric connectivity (Andrews et al., 2006). Homozygous deletions of *Robo1* in the mouse are also associated with occasional heterotopias [ Anthoni et al., 2012; also see review by Gonda et al. (2020)]. Two other genes, *TTRAP* and *THEM2*, are part of the dyslexia risk haplotype that also contains *KIAA0319* on chromosome 6p22.2 and are often included on a list of dyslexia-risk genes. However, the risk haplotype is associated with decreased expression of *KIAA0319*, but not *TTRAP* or *THEM2* (Paracchini et al., 2006). The aromatase gene *CYP19A1* has also been linked to dyslexia (Anthoni et al., 2012), which is interesting, as aromatase determines the conversion of testosterone to estradiol, two sex steroids, and most studies have shown that there is a significant and substantial difference in the prevalence of dyslexia between boys and girls^10^. Neuron specific aromatase has a role in synaptic plasticity and cognitive function in both mouse sexes, and more work is needed to differentiate its effects in males and females (Lu et al., 2019). Expression of *CYP19A1* correlates with expression of dyslexia-risk genes *DYX1C1* and *ROBO1* raising questions as to whether *CYP19A1* acts independently on dyslexia risk. Aromatase has effects on dendritic growth, so an independent role is not excluded, even if not directly proven at present. A study involving Finnish families and an independent study of German families identified a haplotype containing co-regulated genes *C2orf3* and *MRPL19* on chromosome 2p12. The expression of these genes, but not of *FLJ13391* (also in the haplotype) was correlated with the expression of genes *DYX1C1, ROBO1, DCDC2* and *KIAA0319* (Anthoni et al., 2007). No association was found for these genes in a study of Indian families, nor for *ROBO1* or *THEM2* (Venkatesh et al., 2013). Additional animal studies would be useful here to understand the molecular pathways involved and the effects of downregulating the expression of these candidate genes, better to understand possible links to dyslexia.

### Genetics and behavior

Most of the studies linking gene and behavior in dyslexia have been performed in humans, often together with *in vivo* functional brain imaging or neurophysiology to link to brain anatomy and/or behavior. Although such studies are good for establishing correlations, and language and reading can be explored directly, it is much more difficult to make statements about first events and causation. So, is what we learn from those studies something about the cause of the dyslexia or a reflection of the reading problem after years of brain plasticity? A partial answer can be obtained by looking for a phenotype in the youngest person possible to study [see, for example, the work of Gaab and colleagues (Raschle et al., 2011)].^11^ Animal studies, which permit manipulation of genes or the brain in ways not possible in human beings, are superior for looking at the earliest events and for establishing causation, even in the face of the limitations of animal research discussed in the introduction. In fact, animal studies can help differentiate between causal events and subsequent plasticity changes.

Male rats with bilateral freezing lesions to the cortex, which develop focal microgyria, exhibit difficulties in discriminating two sequential tones that occur 332 msecs or less from each other. Male rats with unilateral induction of microgyria are abnormal at a shorter gap, 249 msecs, compared to control animals with sham interventions (Fitch et al., 1994, 1997; Clark et al., 2000a, b). Female rats exposed to the same treatments failed to show a reduced capacity to distinguish rapidly changing sounds (Clark et al., 2000b), even though quantitative analysis of the anatomical changes in the cortex did not disclose any sex differences. Therefore, female rats appear to be more resistant to the behavioral effects of early brain damage in this specific domain, which in turn may help explain sex differences in the incidence of dyslexia and other neurodevelopmental disorders in humans (Krafnick and Evans, 2019; Romeo et al., 2022). In other words, it is not necessarily the case that females are at a lesser risk of exposure to the causal event, but rather they are more likely to react adaptively compared to the males. The cortical microgyria were not different between the sexes, but plasticity effects differed between male and female rats, with males, but not females, showing a shift toward more small neurons (slow neurons?; Goriounova et al., 2018) in the medial geniculate (auditory) nucleus of the thalamus. This raised the question of whether the thalamus, but not the cortex, is critical for acoustic gap detection (see, *c.f.*, Díaz et al., 2012). Thus, an important benefit of the animal model can be to expand the thinking about the mechanisms involved in dyslexia deficits to subcortical areas, while placing less emphasis on the cerebral cortex. The subcortex is important for skill acquisition (Chen et al., 2021), and there is evidence for involvement of the subcortex, including the brainstem, in dyslexia (Hornickel and Kraus, 2013). In the case where a developmental cognitive disorder implicates both the cerebral cortex and subcortical stations, another benefit of animal models would be to help determine whether the problem begins in the subcortex and spread to the cortex, it starts at multiple sites at the same time, or whether the subcortex represents a secondary change following disruption of cortical development. In the latter case, the plasticity, and not the initial change in the cortex, would be responsible for the deficit. It is possible currently to conditionally delete a gene at a selective location, and at a particular time, to help answer this question. In the case of the freezing lesion induced cortical malformation, unpublished results in rats with freezing lesions showed cell composition changes in the thalamus, but also in the cochlear nucleus in the brainstem, again suggesting that the spread from the induced cortical malformation can reach the earliest stages of auditory representation in the central nervous system. It is much more unlikely that a pathology beginning in the brainstem can developmentally propagate rostrad and lead to neuronal migration abnormalities, although brainstem pathology can certainly lead to functional changes in the cortex. Malformations can, however, arise in the brainstem and cortex at the same time (Barkovich, 2012), but we did not see brainstem malformations in the human cases or in any of the animal models that we have used.

The first behavioral genetic model we tried was in rats, with which our collaborators had extensive experience in studying behavior. The choice of the rat as an experimental model had to do with its more extensive behavioral repertoire than the mouse, and because at that time no knockouts were available in mice. The rats had their dyslexia gene homologs suppressed by transfecting with short hairpin RNA interfering constructs. The first gene we suppressed was *Dyx1c1.* The intervention, which caused focal heterotopias, led to deficits in detecting complex auditory stimuli over time (Threlkeld et al., 2007). Auditory processing deficits were seen in male and female rats (Szalkowski et al., 2013). In addition, those animals that also showed heterotopias in hippocampus had deficits in spatial learning (Threlkeld et al., 2007). Additional subtle, but persistent, working memory deficits were demonstrated in Sprague-Dawley rats suppressed with shRNAi to *Dyx1c1* (Szalkowski et al., 2011). In a subsequent study, *Dyx1c1* suppression in rats, in addition to acoustic processing deficits, impaired visual attention in males, without changes in total cortical volume, hippocampal volume, mid-sagittal callosal volume. On the other hand, there were significant changes in the medial geniculate nucleus, with a switch to greater proportions of smaller neurons (Szalkowski et al., 2013).

As with *Dyx1c1*, *in utero* suppression of *Kiaa0319* in rats produces deficits in speech sound discrimination. The experimental animals needed twice as much training in quiet conditions to perform at control levels and remained impaired at several speech tasks (Centanni et al., 2014a). Training using modified speech sounds was able to normalize speech discrimination and physiology (Centanni et al., 2014a). In a separate experiment, the authors reported that with reduced *Kiaa0319* intracellular recordings from affected neurons showed increased neural excitability and input resistance (Centanni et al., 2014b). shRNAi-mediated knockdown of the homolog of the dyslexia risk gene *DCDC2* in the rat resulted in impaired speech sound discrimination without abnormal responses to sound in the primary auditory cortex (Centanni et al., 2016). These results contrasted with those found in *Kiaa031*9 RNAi, which degrades cortical activity to speech sound (Centanni et al., 2014a). The authors emphasized that different dyslexia risk genes affect the speech processing circuits differently. These deficits could not be confirmed in knockout mice for *Kiaa0319*, but double knockout of *Kiaa0319* and *Kiaa0319l* resulted in deficits in central and peripheral auditory function. Deletion of *Kiaa0319l* alone caused abnormalities in the brainstem acoustic wave (Guidi et al., 2017). This is interesting, because brainstem acoustic responses have been documented to be abnormal in dyslexic individuals (Hornickel and Kraus, 2013), and unpublished findings from our laboratory documented abnormalities in neuronal composition in the human and rodent cochlear nucleus.

## Discussion and suggestions for future research

It is clear that animal models offer a limited, albeit important contribution to the understanding of reading disorders, even as such disorders affect only human beings. Endophenotypes such as developmental cortical anomalies and cerebral asymmetries are amenable to modeling even in rodents, as are behavioral endophenotypes involving functional lateralization, sound processing and visual perception. At the cellular level, neuronal hyperexcitability and abnormalities of cilia structure and function occur from dysfunction of dyslexia risk genes in humans and animals. Yet, despite the demonstrated value of animal work, most of the currently funded dyslexia research focuses on human behavior and brain imaging. The value of such research is not in question, but the approaches cannot get directly at the cause of the problem, and therefore cannot link up to powerful available methods for prevention and treatment.

Another limitation of the current human research is its almost exclusive focus on cortical anatomy and physiology and its accompanying behaviors. Thus, although the cerebral cortex is important for language function in adults, and dyslexia in most cases implicates language function, language acquisition requires hearing the sounds of the native language (the congenitally deaf excluded), which begins in infancy (or even *in utero*), and which depends on lower level acoustic processing taking place in the thalamus and brainstem. In the end, if corrupted signals reach the cortex, language can develop abnormally. For speech signals to arrive in the cortex normally, an intact auditory brainstem and thalamus is required, and there is evidence, both from human anatomy and dyslexia animal models, that this may not be the case in dyslexia Tschentscher et al. (2019). That said, *in vivo* imaging the anatomy and function of the human brainstem at the resolution level implicated by the microanatomical studies remains a challenge that relatively few investigators tackle (Tracey and Iannetti, 2006; Beissner et al., 2014; Adil et al., 2021; Lechanoine et al., 2021). Furthermore, although abnormalities in acoustic brainstem physiology in dyslexia has been amply documented (Hornickel and Kraus, 2013; White-Schwoch et al., 2015; Neef et al., 2017), interest in the brainstem’s role during early development in the risk for dyslexia has not grown as it should. In the end, even if it is this cortical dysfunction that accounts for the core symptoms in dyslexia, it is important to know how that dysfunction arose and how to prevent it. Here is an area where animal models can be particularly useful.

Genes that provide increased risk for dyslexia are expressed widely in the brain. But, looking at the pattern of expression alone does not provide useful information for figuring out what is going on. So, for instance, if the expression of an anomalous gene leads to increase noise in neural responses to stimuli, it is not likely that this will affect all higher-level functions equally, but rather only those functions that require precise timing, e.g., phonological processing. In other words, hitting neurons that are a part of systems that do not deal with precise timing will not produce noticeable changes in behaviors. The acoustic system is one of the fastest processors in the brain, if not the fastest. It has to be capable of representing stimuli that differ from each other by only a few milliseconds. This is the sort of difference that distinguishes the sound/b/from the sound/p/. Failure to do this may lead to degraded representations of both sounds and thus introduce an additional difficulty for mapping a sound to a letter while attempting to read. Young readers depend much more on this ability in order to read, since adults eventually graduate from letter by letter reading when they are capable of using efficient top-down mechanisms to divine the word without actually having to read it (unless it is a new word or the context is ambiguous and unhelpful). In fact, it is quite clear that those dyslexics who compensate for their earlier reading difficulties do it by relying of top-down, executive processes that avoid having to decode words letter by letter. A corollary would be that dyslexics who cannot compensate as they grow may suffer from executive dysfunction (Brosnan et al., 2016; Smith-Park et al., 2016).

The emphasis on subcortical system concerns the origin of the dyslexia risk in the brain. Developmental plasticity dictates that secondary and further changes will occur in other parts of the brain as a result of the initial event, downstream of the acoustic stimuli, part of a flexible interconnected network. Dysfunction in one node in this network can reroute connections and reframe the network’s topography and function. With this type of reorganization under adversity, some compensation for loss of function may emerge, but worsening is a real possibility too. In fact, developmental plasticity did not evolve to reformat a network after a lesion in one or more of its nodes, but rather for learning and growth. When these plasticity mechanisms are summoned to fix a big problem, a pathological event, it should not be expected that they will work well. In fact, more often than not they make matters worse. Thus, as an infant with a genetic risk for dyslexia grows, eventually the cortex may show cortical reorganization (disorganization?) in its language networks. Imaging and other approaches to demonstrating cortical organization for language will be aberrant, but is that the cause of the reading disorder? Perhaps, it is the immediately proximal cause, but the problem is just as likely not to have started there, but instead at nodes closer to the sensory (in this case acoustic) input. A goal of prevention would be to address the phenomena that are happening earliest in development. And, for as long as it remains out of reach to test and manipulate these early nodes in babies and infants, the use of animal models is crucial for shedding light on those early events.

Nothing has been said in this paper about visual causes of dyslexia. This author believes that visual causes exist, and in fact, he has come in contact with individuals whose dyslexia was visual, without a doubt (see, for instance, Vannuscorps et al., 2021). However, it is likely that visual causes of dyslexia alone are uncommon compared to those of acoustic origin, and it is possible that they affect dyslexic women more often than men (the few cases seen by the author have all been women). Recall also that the limited published neuropathological findings in dyslexic women were different from those of the typical dyslexic man (Humphreys et al., 1990). However, these statements are made in a most tentative manner and are meant mainly to encourage research on sex difference in the causes, brain findings, and cognitive profiles of dyslexia.

## Author contributions

The author confirms being the sole contributor of this work and has approved it for publication.

## Footnotes

1 Typically, a measurable, relatively simple biological marker lying between a genomic sequence and a complex behavioral disorder, whereby the marker is likely to predict for the presence of the disorder [coined in the 1970s by U.S. behavioral geneticist and clinical psychologist Irving I. Gottesman (1930–2016) – and British psychiatrist James Shields (1918 – 1978) (Gottesman and Shields, 1972). In animal models an endophenotype must be present in the animal and in the human.

2 Considering the different neuropathology described in men and women with dyslexia, as well as the Altarelli et al. (2014) finding of normal patterns of planum asymmetry in the women and girls in their study, could it be that dyslexia is a different disorder in men and women?

3 This is also the case for rats undergoing shRNAi experiments and for mice with gene deletions.

4 The term plasticity often conjures up the notion of recovery after injury (Kolb, 2003), but positive outcomes of plasticity do not necessarily follow (Johnston, 2004).

5 By convention, human genes are italicized in all caps; mouse genes are italicized with only the first letter in cap; proteins for both humans and animals are in all caps without italics.

6
https://www.ncbi.nlm.nih.gov/gene/161582

7
https://www.uniprot.org/uniprot/Q5DU00

8
https://www.proteinatlas.org/ENSG00000137261-KIAA0319

9
https://www.uniprot.org/uniprot/Q5SZV5

10 This finding implicates a sex hormone effect. Not counting substantial social and environmental influences, there exist sex differences based on the presence or absence of the y chromosome, but this would dictate that the phenotype be present only in boys and men, e.g., ear hair.

11 “Partial answer” refers to the fact that, although anatomical differences can be imaged before the onset of reading, which indicates that they are not caused by reading differences, the imaging cannot tell how early the differences arise, which is likely to be *in utero* and before the onset of speech itself.
